# Computational approaches: discovery of GTPase HRas as prospective drug target for 1,3-diazine scaffolds

**DOI:** 10.1186/s13065-019-0613-8

**Published:** 2019-07-24

**Authors:** Sanjiv Kumar, Deepika Sharma, Balasubramanian Narasimhan, Kalavathy Ramasamy, Syed Adnan Ali Shah, Siong Meng Lim, Vasudevan Mani

**Affiliations:** 1 Faculty of Pharmaceutical Sciences, Maharshi Dayanand University, Rohtak, 124001 India; 2 Faculty of Pharmacy, Universiti Teknologi MARA (UiTM), 42300 Bandar Puncak Alam, Selangor Darul Ehsan Malaysia; 3 Collaborative Drug Discovery Research (CDDR) Group, Pharmaceutical Life Sciences Community of Research, Universiti Teknologi MARA (UiTM), 40450 Shah Alam, Selangor Darul Ehsan Malaysia; 4 Atta-ur-Rahman Institute for Natural Products Discovery (AuRIns), Universiti Teknologi MARA, Puncak Alam Campus, 42300 Bandar Puncak Alam, Selangor Darul Ehsan Malaysia; 5 Department of Pharmacology and Toxicology, College of Pharmacy, Qassim University, Buraidah, 51452 Kingdom of Saudi Arabia

**Keywords:** PharmMapper, 1,3-Diazines, GTPase HRas, Docking, HCT116 cancer cell

## Abstract

Heterocyclic 1,3-diazine nucleus is a valuable pharmacophore in the field of medicinal chemistry and exhibit a wide spectrum of biological activities. PharmMapper, a robust online tool used for establishing the target proteins based on reverse pharmacophore mapping. PharmMapper study is carried out to explore the pharmacological activity of 1,3-diazine derivatives using reverse docking program. PharmMapper, an open web server was used to recognize for all the feasible target proteins for the developed compounds through reverse pharmacophore mapping. The results were analyzed via molecular docking with maestro *v11.5* (Schrodinger 2018-1) using GTPase HRas as possible target. The molecular docking studies displayed the binding behavior of 1,3-diazine within GTP binding pocket. From the docking study compounds **s3** and **s14** showed better docked score with anticancer potency against cancer cell line (HCT116). Hence, the GTPase HRas may be the possible target of 1,3-diazine derivatives for their anticancer activity where the retrieved information may be quite useful for developing rational drug designing. Furthermore the selected 1,3-diazine compounds were evaluated for their in vitro anticancer activity against murine macrophages cell line. 1,3-Diazine compounds exhibited good selectivity of the compounds towards the human colorectal carcinoma cell line instead of the murine macrophages. The toxicity study of the most active compounds was also performed on non cancerous HEK-293 cell line.

**Electronic supplementary material:**

The online version of this article (10.1186/s13065-019-0613-8) contains supplementary material, which is available to authorized users.

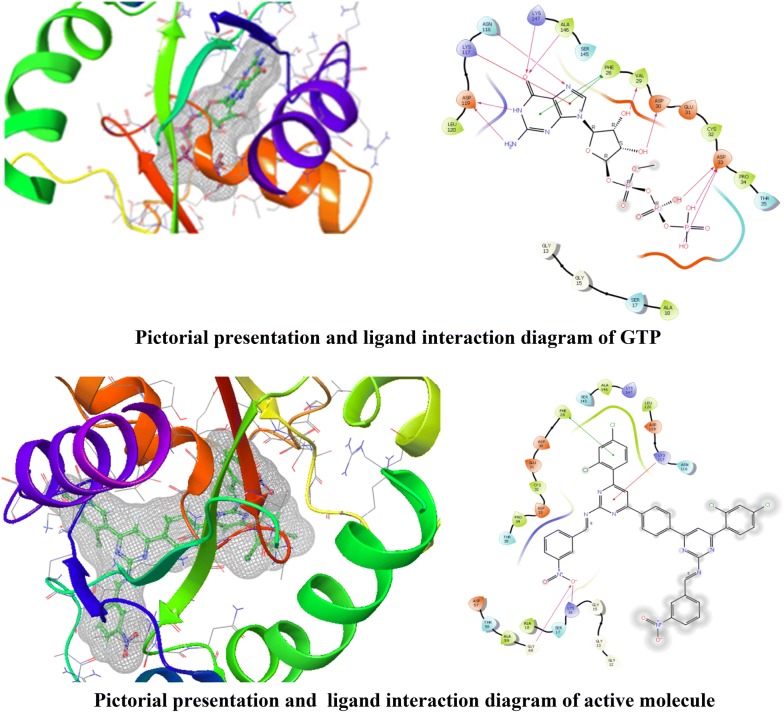

## Introduction

Heterocyclic compounds play the vital role in pharmaceutical field due to their specific chemical reactivity and block the normal functioning of biological receptors. A large number of 1,3-diazine derivatives are reported to exhibit various biological activities i.e. anticancer [1], antibacterial [2], anti-inflammatory, analgesic [3], antimicrobial activity [4]. 1,3-Diazine nucleus is the building unit in DNA and RNA thus 1,3-diazine based compounds exhibit diverse biological activities. Thus 1,3-diazine and its derivative attract the researchers to further explore their biological activities [5].

According to World Health Organization (WHO) reports, cancer is one of the leading causes of death worldwide and is projected to continuously rising, with approximately 11.5 million deaths in 2030. The main types of cancer are of body organs like lung, stomach, colorectal, liver and breast. Cancer treatment includes psychosocial support, surgery, radiotherapy, chemotherapy that is aimed at curbing the disease as well as improving the quality of patient’s life [6]. Malignancy arises due to transformation of the genetic material of a normal cell, followed by successive mutations, ultimately leading to the uncontrolled division of cells. Drug resistance is a phenomenon that results when diseases become tolerant to pharmaceutical treatments. Drug resistance occurs through various mechanisms like drug inactivation, drug target alteration, drug efflux, DNA damage repair, cell death inhibition [7].

In modern drug discovery, molecular docking is now a day’s an advanced computational technique used to study the ligand–receptor interactions using docking software and uses conformational and electrostatic interactions to measures it. Molecular docking programs perform a search algorithm in which the conformation of the ligand is evaluated until the convergence to the minimum energy is reached. With the various docking strategies, the ligand specificity against a particular target (receptor) can be calculated computationally in which best fitting ligand can be used for further lead optimization process. The docking score (affinity scoring function), ΔG [U total in kcal/mol], is the sum of the electrostatic and van der Waals energies to rank the candidate poses to determine their binding potentialities. Docking score is calculated terms of negative energy [8, 9]. The heterocyclic pyrimidine derivatives displayed good anticancer potency against HCT116 cancer cell line [10–12].

Based on the above mentioned facts, in the present study, the reverse docking program was used to recognize the drug target for the anticancer activity of 1,3-diazine derivatives (identified in an earlier study [13]) using PharmMapper web server application tool. The receptor (target), GTPase HRas was found with good fitness score against cancer. The validation of the indicated target was done with molecular docking using maestro *v11.5.*

## Experimental

### Data set

**Scheme 1:**
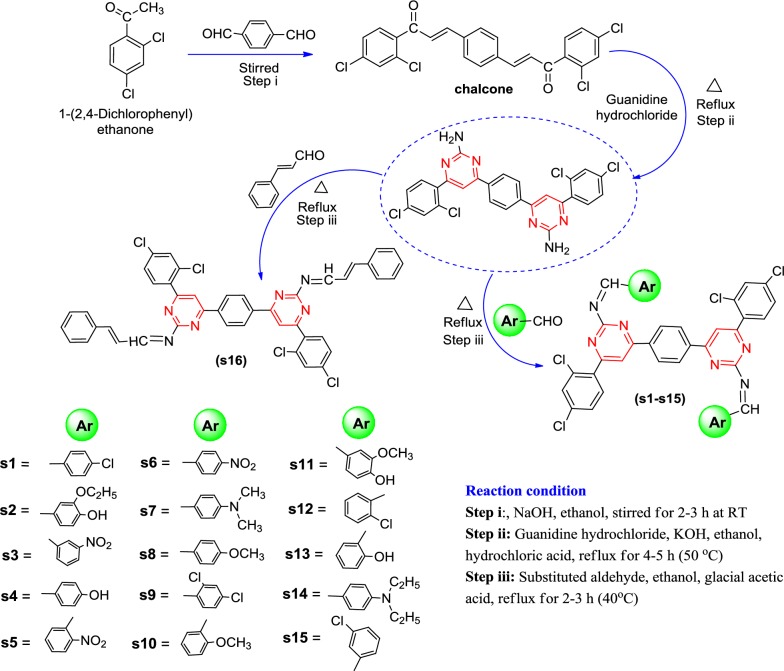
Synthetic scheme for the synthesized 1,3-diazine derivatives (**s1**–**s16**)

The data set of reported 1,3-diazine derivatives (**s1**–**s16**) have good anticancer activity against human colorectal carcinoma cancer cell line (HCT116) were selected from the earlier study for the establishment of the pharmacophore model. The synthesis of the reported compounds is shown in synthetic Scheme 1. The physicochemical properties and structural elucidations are shown in Tables 1 and 2, respectively. The molecular structures of the selected data set of 1,3-diazine derivatives and their anticancer screening results are shown in Table 3 [13].

**Table 1:** Physicochemical properties of the selected data set (1,3-diazine derivatives)

Comp. no	Molecular formula	Color	R_*f*_ value	m.pt. °C	% Yield
**s1.**	C_40_H_22_Cl_6_N_6_	Dark yellow	0.46	133–135	85.45
**s2.**	C_44_H_32_Cl_4_N_6_O_4_	Light yellow	0.25	113–115	75.56
**s3.**	C_40_H_22_Cl_4_N_8_O_4_	Cream yellow	0.31	140–142	69.03
**s4.**	C_40_H_24_Cl_4_N_6_O_2_	Pure yellow	0.26	133–135	82.56
**s5.**	C_40_H_22_Cl_4_N_8_O_4_	Medallion yellow	0.35	146–148	70.00
**s6.**	C_40_H_22_Cl_4_N_8_O_4_	Light yellow	0.32	142–144	75.65
**s7.**	C_44_H_34_Cl_4_N_8_	Light yellow	0.39	123–125	78.12
**s8.**	C_42_H_28_Cl_4_N_6_O_2_	Pure yellow	0.23	124–126	80.45
**s9.**	C_40_H_20_Cl_8_N_6_	Lemon yellow	0.21	80–82	79.34
**s10.**	C_42_H_28_Cl_4_N_6_O_2_	Light yellow	0.58	134–135	82.23
**s11.**	C_42_H_28_Cl_4_N_6_O_4_	Pure yellow	0.41	129–131	89.45
**s12.**	C_40_H_22_Cl_6_N_6_	Medallion yellow	0.43	56–58	85.56
**s13.**	C_40_H_24_Cl_4_N_6_O_2_	Dark yellow	0.50	79–81	87.23
**s15.**	C_48_H_42_Cl_4_N_8_	Cream yellow	0.37	75–77	66.33
**s15.**	C_40_H_22_Cl_6_N_6_	Dark yellow	0.57	56–58	68.12
**s16.**	C_44_H_28_Cl_4_N_6_	Light yellow	0.50	63–65	62.23

**Table 2:** Structural elucidation data of the selected data set (1,3-diazine derivatives)

Comp.	IR KBr (cm^−1^)						^1^H-NMR (DMSO-*d*_6_, ppm)	^13^C-NMR (DMSO-*d*_6_, ppm)	Elemental analysis (CHN); MS: *m/z* (M^+^+1)
	C–H	C=C str.	C–N str.	N=CH str.	C–Cl str.	Other			
**s1**	3088.9	1596.0	1332.1	1665.8	735.7	–	7.36–7.86 (m, 18H, Ar–H), 10.0 (s, 2H, N=CH), 10.2 (s, 2H, (CH)_2_ of pyrimidine ring)	160.2, 145.7, 138.9, 136.2, 131.2, 130.2, 129.9, 129.9, 128.1, 128.1, 127.6	Theoretical calc: C, 60.10; H, 2.77; N, 10.51; Found: C, 60.18; H, 2.71; N, 10.56; 797
**s2**	2978.5	1595.2	1331.5	1665.8	736.6	1104.4 (C–O–C_2_H_5_, aralkyl ether), 3345.0 (O–H str.)	6.78–8.27 (m, 16H, Ar–H), 9.70 (s, 2H, N=CH), 10.03 (s, 2H, (CH)_2_ of pyrimidine ring), 3.98 (t, 4H, (CH_2_)_2_), 1.32 (d, 6H, (CH_3_)_2_)	165.6, 164.0, 153.5, 147.6, 136.6, 137.1, 132.6, 132.5, 130.4, 129.4, 129.9, 128.8, 126.4, 112.4, 107.3, 64.4, 14.8	Theoretical calc: C, 62.13; H, 3.79; N, 9.88; Found: C, 62.10; H, 3.68; N, 9.81; 849
**s3**	3088.0	1595.5	1337.0	1665.3	734.2	1373.8 (C–NO_2_, str., NO_2_)	7.42–8.34 (m, 18H, Ar–H), 10.04 (s, 2H, N=CH), 10.15 (s, 2H, (CH)_2_ of pyrimidine ring)	165.5, 164.3, 145.9, 138.9, 137.8, 137.4, 136.2, 135.3, 131.9, 130.4, 129.4, 129.8, 128.2, 127.6, 124.4	Theoretical calc: C, 58.56; H, 2.70; N, 13.66; Found: C, 58.50; H, 2.73; N, 13.70; 819
**s4**	3028.0	1595.4	1332.8	1664.0	735.8	3461.3 (O–H str.)	7.33–8.34 (m, 18H, Ar–H), 9.79 (s, 2H, N=CH), 10.10 (s, 2H, (CH)_2_ of pyrimidine ring), 6.92 (s, 2H, Ar–OH)	165.5, 164.3, 136.4, 136.4, 134.9, 130.2, 130.1, 129.9, 129.0, 128.0, 127.6, 116.3, 107.2	Theoretical calc: C, 63.01; H, 3.17; N, 11.02; Found: C, 63.05; H, 3.19; N, 11.08; 761
**s5**	3028.7	1595.1	1333.1	1664.5	736.4	1372.9 (C–NO_2_ sym. str., NO_2_)	7.02–8.34 (m, 18H, Ar–H), 10.0 (s, 2H, N=CH), 10.10 (s, 2H, (CH)_2_ of pyrimidine ring)	165.5, 164.3, 142.3, 137.8, 134.9, 132.6, 131.3, 130.2, 130.2, 129.9, 129.9, 128.1, 128.1, 127.6, 107.2	Theoretical calc: C, 58.56; H, 2.70; N, 13.66; Found: C, 58.60; H, 2.74; N, 13.69; 819
**s6**	3088.0	1595.4	1334.0	1664.0	735.8	1372.5 (C–NO_2_ sym. str., NO_2_)	7.02–8.34 (m, 18H, Ar–H), 10.0 (s, 2H, N=CH), 10.1 (s, 2H, (CH)_2_ of pyrimidine ring)	165.5, 164.3, 163.1, 145.8, 142.7, 140.0, 137.8, 136.9, 134.9, 132.7, 131.8, 130.2, 129.9, 128.1, 124.7, 107.1	Theoretical calc: C, 58.56; H, 2.70; N, 13.66; Found: C, 58.61; H, 2.76; N, 13.70; 819
**s7**	3028.0	1590.1	1331.9	1662.8	733.8	2826.1 (C–H str., –CH_3_)	6.77–8.34 (m, 18H, Ar–H), 9.67 (s, 2H, N=CH), 10.04 (s, 2H, (CH)_2_ of pyrimidine ring), 3.04 (s, 12H, (CH_3_)_4_)	165.5, 164.3, 145.7, 142.7, 137.8, 136.4, 134.9, 131.3, 130.2, 130.1, 129.9, 128.1, 127.6, 111.5, 107.2, 40.1	Theoretical calc: C, 64.72; H, 4.20; N, 13.72; Found: C, 64.76; H, 4.26; N, 13.74; 815
**s8**	3027.8	1595.3	1332.4	1664.3	736.3	3088.9 (C–O–CH_3_, aralkyl ether)	7.02–8.34 (m, 18H, Ar–H), 10.05 (s, 2H, N=CH), 10.10 (s, 2H, (CH)_2_ of pyrimidine ring), 3.84 (s, 6H, (OCH_3_)_2_)	165.5, 164.3, 163.5, 145.8, 139.9, 134.9, 132.7, 130.4, 129.8, 128.1, 128.1, 127.9, 114.9, 108.0, 52.2	Theoretical calc: C, 63.81; H, 3.57; N, 10.63; Found: C, 63.85; H, 3.61; N, 10.70; 789
**s9**	3025.9	1594.6	1330.9	1663.1	734.9	–	7.28–8.02 (m, 16H, Ar–H), 10.02 (s, 2H, N=CH), 10.10 (s, 2H, (CH)_2_ of pyrimidine ring)	165.5, 164.3, 163.5, 145.8, 137.8, 136.9, 137.7, 131.7, 130.9, 130.2, 129.8, 128.0, 128.0, 127.5, 100.0	Theoretical calc: C, 55.33; H, 2.32; N, 9.68; Found: C, 55.338; H, 2.37; N, 9.72; 865
**s10**	3027.4	1592.1	1332.0	1663.4	735.9	3088.5 (C–O–CH_3_, aralkyl ether)	6.96–7.87 (m, 18H, Ar–H), 9.20 (s, 2H, N=CH), 10.02 (s, 2H, (CH)_2_ of pyrimidine ring), 3.85 {s, 6H, (OCH_3_)_2_}	165.5, 164.3, 163.5, 145.8, 139.9, 135.9, 134.9, 132.7, 130.4, 129.8, 128.1, 128.1, 127.9, 115.9, 108.0, 55.2	Theoretical calc: C, 63.81; H, 3.57; N, 10.63; Found: C, 63.85; H, 3.61; N, 10.68; 789
**s11**	3027.2	1596.3	1331.3	1663.9	736.0	3461.4 (O–H str.), 3088.4 (C–O–CH_3_, aralkyl ether)	7.03–8.34 (m, 16H, Ar–H), 10.04 (s, 2H, N=CH), 10.10 (s, 2H, (CH)_2_ of pyrimidine ring), 3.85{s, 6H, (OCH_3_)_2_}	165.5, 164.3, 151.0, 149.0, 137.7, 136.9, 134.9, 132.7, 131.8, 130.9, 130.3, 129.8, 128.1, 128.0, 107.6, 61.3	Theoretical calc: C, 61.33; H, 3.43; N, 10.22; Found: C, 61.38; H, 3.48; N, 10.27; 821
**s12**	2973.4	1599.6	1329.1	1666.7	750.4	–	7.24–8.00 (m, 18H, Ar–H), 9.01 (s, 2H, N=CH), 10.10 (s, 2H, (CH)_2_ of pyrimidine ring)	165.6, 164.3, 162.5, 146.7, 136.2, 134.3, 131.8, 130.3, 130.2, 130.1, 129.9, 129.2, 120.0, 128.0, 127.8, 100.3	Theoretical calc: C, 60.10; H, 2.77; N, 10.51; Found: C, 60.17; H, 2.80; N, 10.55; 797
**s13**	2972.9	1598.7	1330.1	1698.9	750.5	3360.9 (O–H str.)	7.27–7.99 (m, 18H, Ar–H), 9.99 (s, 2H, N=CH), 10.07 (s, 2H, (CH)_2_ of pyrimidine ring)	165.5, 164.3, 137.7, 136.4, 130.3, 129.9, 128.9, 128.1, 117.0, 110.0	Theoretical calc: C, 63.01; H, 3.17; N, 11.02; Found: C, 63.05; H, 3.19; N, 11.07; 761
**s14**	2974.0	1590.9	1352.3	1695.1	750.1	–	7.26–8.00 (m, 18H, Ar–H), 9.63 (s, 2H, N=CH), 10.0 (s, 2H, (CH)_2_ of pyrimidine ring), 3.38–3.49 {q, 8H, (CH_2_)_4_}, 1.07–1.15 {t, 12H, (CH_3_)_4_}	167.5, 164.3, 159.3, 136.4, 131.8, 130.3, 130.1, 129.9, 128.9, 127.4, 126.6, 124.4, 111.0, 44.4, 12.7	Theoretical calc: C, 66.06; H, 4.85; N, 12.84; Found: C, 66.10; H, 4.90; N, 12.88; 871
**s15**	2974.4	1579.0	1328.3	1693.2	750.1	–	7.25–8.03 (m, 18H, Ar–H), 10.00 (s, 2H, N=CH), 10.04 (s, 2H, (CH)_2_ of pyrimidine ring)	165.5, 164.3, 136.9, 131.8, 131.7, 130.3, 130.2, 129.9, 129.2, 128.9, 128.1, 127.4, 126.6, 100.9	Theoretical calc: C, 60.10; H, 2.77; N, 10.51; Found: C, 60.15; H, 2.80; N, 10.48; 797
**s16**	2973.4	1597.1	1329.5	1669.7	749.8	–	7.45–8.04 (m, 20H, Ar–H), 7.55 {d, 2H, (CH)_2_ of N=CH}, 9.00 (s, 2H, (CH)_2_ of pyrimidine ring), 6.86 {t, 2H, (CH)_2_}, 7.34 {d, 2H, (CH)_2_};	167.8, 164.2, 163.9, 135.6, 134.6, 133.5, 130.2, 130.8, 129.8, 128.0, 128.3, 127.0, 127.9, 120.1, 110.1	Theoretical calc: C, 67.53; H, 3.61; N, 10.74; Found: C, 67.51; H, 3.68; N, 10.77; 787

**Table 3:** Data set of 1,3-diazine derivatives with their anticancer screening results

Comp.	Compound name	Molecular structure	Anticancer activity IC_50_ (µmol/mL) Cancer cell lines	
			HCT116	RAW264.7
**s1.**	6,6′-(1,4-Phenylene)bis(*N*-(4-chlorobenzylidene)-4-(2,4-dichlorophenyl)pyrimidin-2-amine		11.24 ± 1.3	10.26 ± 2.3
**s2.**	4,4′-(((6,6′-(1,4-Phenylene) bis(4-(2,4-dichlorophenyl) pyrimidine-6,2-diyl))bis-(azanylylidene))bis(methanyl-ylidene))bis(2-ethoxyphenol)		3.95 ± 1.2	3.86 ± 1.3
**s3.**	6,6′-(1,4-Phenylene)bis(4-(2,4-dichlorophenyl)-*N*-(3-nitro-benzylidene)pyrimidin-2-amine)		1.06 ± 0.1	3.13 ± 1.6
**s4.**	4,4′-(((6,6′-(1,4-Phenylene)-bis(4-(2,4-dichlorophenyl)-pyrimidine-6,2-diyl))bis(azanyl-ylidene))bis(methanylylidene))-diphenol		10.56 ± 2.6	9.96 ± 3.2
**s5.**	6,6′-(1,4-Phenylene)bis(4-(2,4-dichlorophenyl)-*N*-(2-nitrobenzylidene)pyrimidin-2-amine)		10.11 ± 2.1	10.02 ± 2.2
**s6.**	6,6′-(1,4-Phenylene)bis(4-(2,4-dichlorophenyl)-*N*-(4-nitrobenzylidene)pyrimidin-2-amine)		5.41 ± 1.3	4.12 ± 2.6
**s7.**	6,6′-(1,4-Phenylene)bis(4-(2,4-dichlorophenyl)-*N*-(4-(dimethylamino)benzylidene)pyrimidin-2-amine)		3.70 ± 1.2	3.41 ± 1.5
**s8.**	6,6′-(1,4-Phenylene)bis(4-(2,4-dichlorophenyl)-*N*-(4-methoxy-benzylidene)pyrimidin-2-amine)		2.96 ± 2.1	2.78 ± 2.3
**s9.**	6,6′-(1,4-Phenylene)bis(*N*-(2,4-dichlorobenzylidene)-4-(2,4-dichlorophenyl)pyrimidin-2-amine)		1.26 ± 1.7	2.61 ± 1.2
**s10.**	6,6′-(1,4-Phenylene)bis(4-(2,4-dichlorophenyl)-*N*-(2-methoxy-benzylidene)pyrimidin-2-amine)		3.23 ± 1.2	2.24 ± 2.2
**s11.**	4,4′-(((6,6′-(1,4-Phenylene) bis(4-(2,4-dichlorophenyl) pyrimidine-6,2-diyl))bis-(azanylylidene))bis(methanyl-ylidene))bis(2-methoxyphenol)		3.04 ± 1.23	1.97 ± 2.3
**s12.**	6,6′-(1,4-Phenylene)bis(*N*-(2-chlorobenzylidene)-4-(2,4-dichlorophenyl)-pyrimidin-2-amine)		2.55 ± 1.2	2.57 ± 1.2
**s13.**	2,2′-(((6,6′-(1,4-Phenyl-ene)bis(4-(2,4-dichloro-phenyl)pyrimidine-6,2-diyl))bis-(azanylylidene))bis(methanyly-lidene))diphenol		1.33 ± 1.3	2.27 ± 1.4
**s14.**	6,6′-(1,4-Phenylene)bis(4-(2,4-dichlorophenyl)-*N*-(4-(diethyl-amino)benzylidene)pyrimidin-2-amine)		1.08 ± 1.1	2.11 ± 1.6
**s15.**	6,6′-(1,4-Phenylene)bis(*N*-(3-chlorobenzylidene)-4-(2,4-dichlorophenyl)-pyrimidin-2-amine)		1.54 ± 1.1	2.62 ± 1.5
**s16.**	4-(2,4-Dichlorophenyl)-6-(4-(6-(2,4-dichlorophenyl)-2-(((*E*)-3-phenylallylidene)-amino)-pyrimidin-4-yl)-phenyl)-*N*-((*E*)-3-phenyl-allylidene)pyrimidin-2-amine		2.39 ± 1.2	1.89 ± 1.7

Data were expressed as the mean ± standard error (SE)

### Ligand preparation

Ligand preparation is done using the maestro *v11.5* LigPrep module to deliver the best results, the docked structures must be good representations of the actual ligand structures as they would appear in a complex of protein–ligand. This implies that the structure must fulfill the following requirements for Glide docking program. They have to be 3-dimensional (3D). Glide only modifies the ligand’s internal torsional coordinates during docking, so the remaining geometric parameters need to be optimized in advance. They must each consist of a single molecule without covalent receptor bonds, with no accompanying fragments, such as counter ions and solvent molecules. They have to be filled with all their hydrogen (valences). For physiological pH values (around 7), they must have a suitable protonation state [14, 15].

### Protein preparation

The protein chosen for the molecular docking study of selected data set of 1,3-diazine derivatives, GTPase HRas (PDB Id: 2CL7) was obtained from the Protein Data Bank (Additional file 1). The typical structure file imported from the PDB is not suitable for immediate use in performing calculations for molecular modeling. A typical PDB structure file consists of heavy atoms and may include a co-crystallized ligand, molecules of water, ions of metal and cofactors. In the protein preparation wizard, protein was prepared where protein was preprocessed, optimized and minimized. The outcome is refined, hydrogenated ligand and ligand–receptor complicated structures that are appropriate for use with other Schrödinger modules [16].

### Grid generation

Maestro *v11.5* receptor grid generation module (Schrodinger 2018-1) is used to generate grid. A grid is generated around the binding site already occupied by the co-crystallized ligand so that it is feasible to exclude co-crystallized ligand and to attach new molecule to the same binding site to study the docking of 1,3-diazine derivatives [17].

### Docking study

After generating the glide grid zip file and preparing the ligands, docking was performed in the maestro *v11.5* glide module. The series of ligands (1,3-diazines) was tested using additional accuracy (XP) via GTP binding site. XP Module conducts more accurate molecular docking of the selected molecules of 1,3-diazine nucleus. The size of the dataset is reduced as the precision of the docking increase at each stage. In the maestro v11.5, the XP parameters such as docking score glide energy and glide emodel value were calculated [18, 19].

### Anticancer evaluation

Anticancer activity of the synthesized compounds was evaluated on the cell line of murine macrophage (RAW 264.7) by SRB assay (Table 3) [20]. The murine macrophage cell line was seeded at 7000 cells/well on the 96 flat bottom well plate and allowed to be activated overnight. Then the cells were exposed for 72 h to the respective compounds and subjected to the SRB test. Then treated cells were located in trichloroacetic acid and stained in SRB dye [0.4% (w/v) mixed with 1% acetic acid]. The plate’s optical density was read with a microplate reader at 570 nm.

### Cell toxicity evaluation

The cell toxicity study of the selected compounds was performed on non-cancer cell line, i.e. human embryonic kidney (HEK 293). In Dulbecco’s modified Eagle medium (10% heat inactivated FBS) human embryonic kidney cells were maintained. Penicillin and streptomycin antibiotics were added and placed in a 5% CO_2_ incubator for colorimetric-based using MTT assay at 37 °C. Compounds **s3**, **s9**, **s13**–**s16** were seeded on a 24-h 96-well plate with five thousand HEK-293 cells (viability 98%). Wells have been added to MTT 5 mg/ml for 4 h after 24 h incubation [21]. Using the Synergy/HTX MultiScan reader (BioTek) absorbance at 580 nm was registered and the lethal dose LD_50_ was calculated and the selectivity index (SI) calculated.

## Results and discussion

### Target recognition

An open web portal, PharmMapper was used through reverse pharmacophore mapping to account for all possible compound targets [22]. PharmMapper sets the feasible potential targets based on the reverse pharmacophore mapping of given 1,3-diazine compounds. It compares the pharmacophores of the compounds given to the BindingDB, TargetBank, DrugBank, PDTD with 16,159 druggable and 51,431 ligandable pharmacophore models in built pharmacophore models database of 23,236 proteins. It offers outcomes in the form of Z score based on the resemblance of pharmacophore of specified compounds with the recognized target pharmacophore model as well as the significance of target protein in illnesses and signs are provided as well [23, 24]. In order to define its potential drug target, the most active compounds **s3** and **s14** were presented to PharmMapper. Depending on their role in cancer initiation and progression, target protein was chosen.

### Target identification

From the information collection, the PharmMapper (http://59.78.96.61/pharmmapper) received compounds **s3** and **s14** showing the advanced anti-carcinogenic activity. The pharmacophores of the potent compounds **s3** and **s14** were compared with the built-in pharmacophore model database. PharmMapper compared the pharmacophores of the potent compounds **s3** and **s14** with the created-in pharmacophore model database and generated 250 protein target information with their fitness score and pharmacophoric characteristics, indication and importance of each protein. 250 Protein retrieved were ranked based on their fitness score. Top five proteins with fitness score more than 5.0 were studied to establish the possible target protein for compounds **s3** and **s14** and target selection was done based upon the role of protein in cancer disease (Table 4). GTPase HRas protein with 15 pharmacophoric characteristics (8 acceptor, 5 donor and 2 negative) scored 5.424 out of the top five proteins, was discovered to play a crucial part in cancer determinism. Another protein with a healthy fitness value, but as shown in Table 4, did not account for any disease. The GTPase HRas protein function is governed by the GTP where GTP becomes GDP. GTP-based HRas protein’s mechanism of action is discovered to function by signal transduction in regulating cell division and cell growth. Mutation in HRas has been shown to lead to different cancer types such as bladder, Costello syndrome, bladder cancer, etc. Because HRas belongs to the oncogene family, healthy cells can become cancerous [25]. GTPase HRas has been further assessed through the docking program for the binding affinity for the studied 1,3-diazine derivatives.

**Table 4:** Details of top five proteins hits from PharmMapper pharmacophore mapping

S. no.	Protein name	PDB id	Disease	No. of pharmacophore features	Fitness score
1.	Aspartate aminotransferase	1ASG	None	9	5.443
2.	Palmitoyl-protein thioesterase 1	1EH5	None	6	5.421
3.	Chorismate synthase	1QXO	None	10	5.323
4.	GTPase HRas	2CL7	Defects in HRAS are the cause of oral squamous cell carcinoma (OSCC), costello syndrome, congenital myopathy, bladder cancer, Hurthle cell thyroid carcinoma, thyroid cancers, tumor redisposition	15	5.424
5.	UPF0230 protein TM_1468	1VPV	None	7	5.822

### Docking

**Fig. 1:**
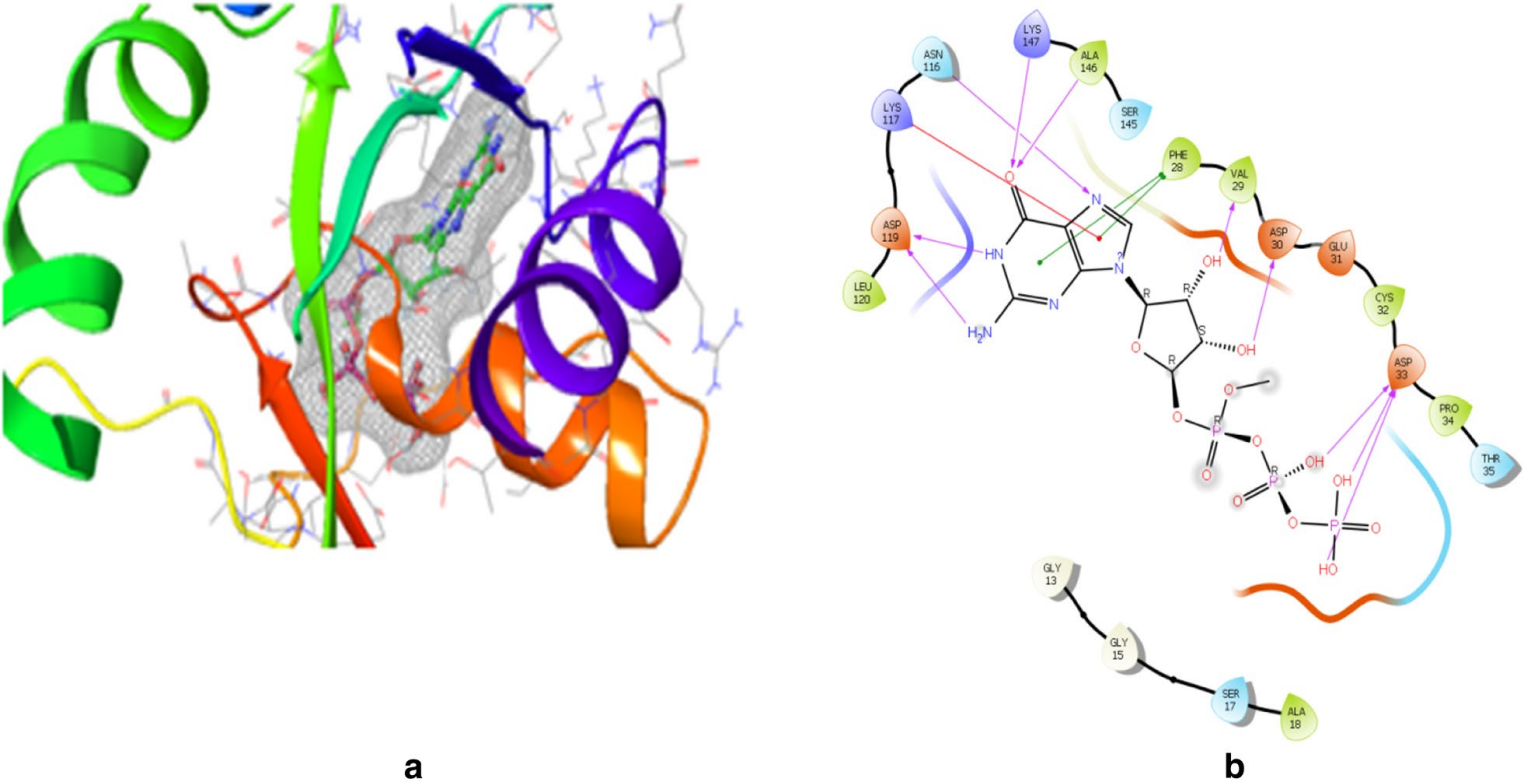
Pictorial presentation 3D (**a**) and ligand interaction diagram 2D (**b**) of GTP

**Fig. 2:**
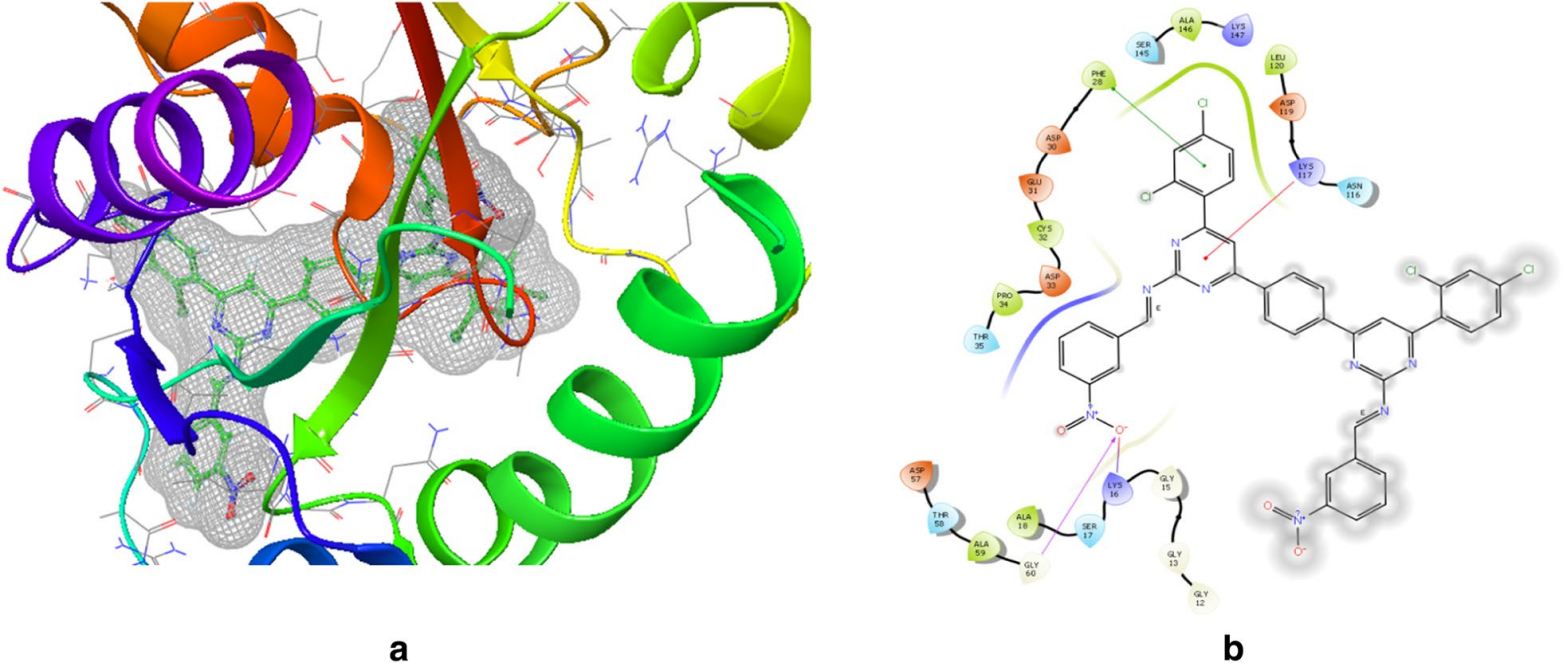
Pictorial presentation 3D (**a**) and ligand interaction diagram 2D (**b**) of compound **s3**

**Fig. 3:**
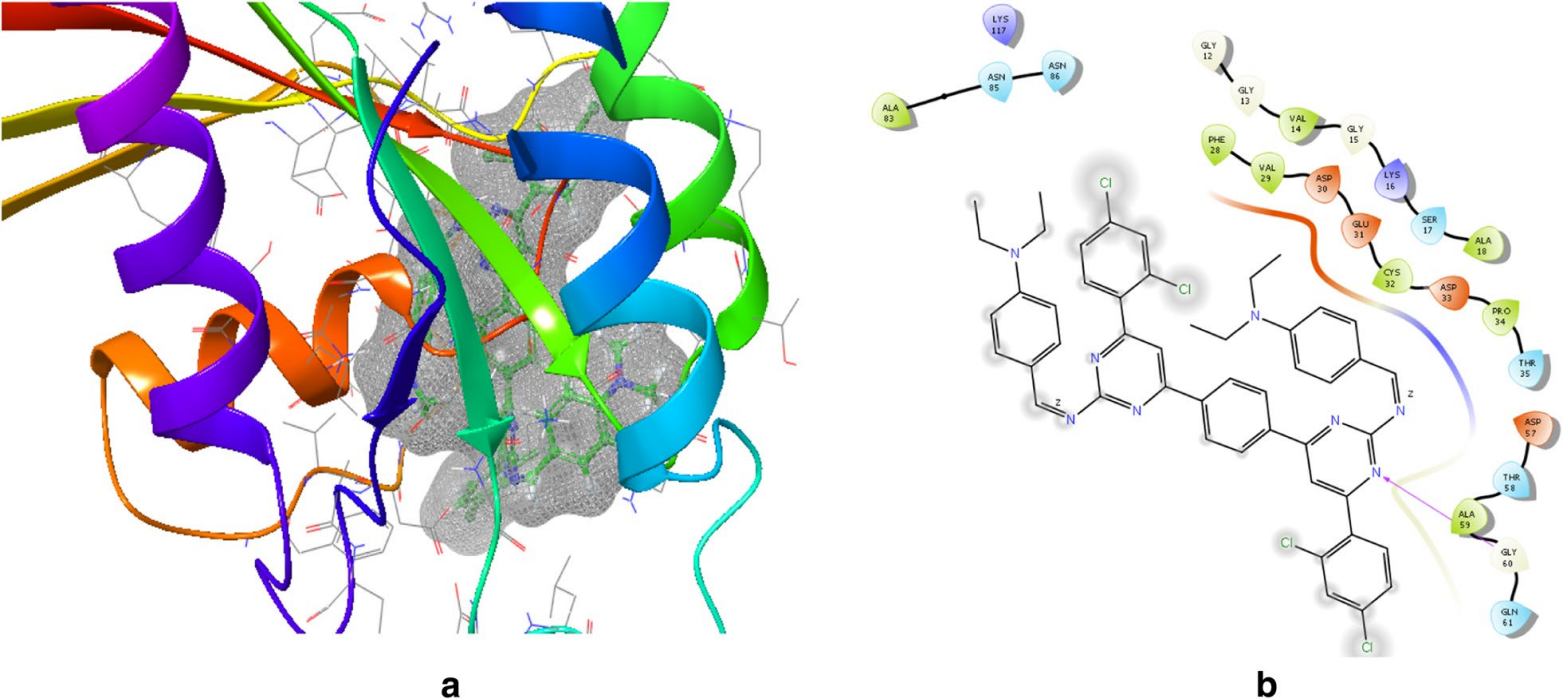
Pictorial presentation 3D (**a**) and ligand interaction diagram 2D (**b**) of compound **s14**

Previously, GTPase HRas and 1,3-diazine derivatives were ready for docking and then docked using maestro *v11.5* Glide module (Schrodinger 2018-1). GTP was maintained as docking control with docked score = − 10.434 and glide energy = − 80.151 in order to score the compounds to be studied. The docking is performed using PDB Id: 2CL7 (Fig. 1) in the same binding region of already co-crystallized GTP ligand. All 1,3-diazine compounds were scored using flexible docking (XP docking) where compounds used GTP as docking control. Minimization of docked compounds was carried out within the binding site and the most stable orientation was analyzed with the lowest possible energy. Docking score of the compounds is shown in Table 5. The results of PharmMapper and molecular docking showed the specificity of 1,3-diazine compounds for the protein GTPase HRas. Compounds demonstrated excellent interaction with GTPase HRas and binding affinity. If we look at the binding mode of most active compounds **s3** and **s14** within the binding region, compound **s3** has docked in the binding pocket score (− 2.14) and glide energy (− 56.46) and hydrogen bond formation with crucial amino acid residue Gly60 with oxygen atom; Compound **s14** has docked score (− 1.603) and glide force (− 66.638) and hydrogen bond formation in the binding pocket with vital amino acid residue Gly60 (Table 6, Figs. 2 and 3). Thus the docking results suggested that the compounds of 1,3-diazine could be of great interest in successful chemotherapy. The GTPase HRas may therefore be the possible target for their anticancer potential of 1,3-diazine derivatives. The experimental research will be carried out to validate the affinity to target protein and the binding mode of inhibition of compounds. The docking results of the data set and GTPas shown in Additional files 2, 3.

**Table 5:** Molecular docking results of 1,3-diazine derivatives (**s1**–**s16**)

Comp.	Docking score	Glide energy (kcal/mol)	Glide emodel
**s1**	− 0.951	− 68.028	− 93.889
**s2**	− 4.195	− 80.703	− 107.394
**s3**	− 2.14	− 56.46	− 75.84
**s4**	− 2.816	− 71.535	− 99.49
**s5**	− 2.316	− 54.01	− 72.719
**s6**	− 1.394	− 78.785	− 112.194
**s7**	− 1.077	− 76.603	− 109.074
**s8**	− 0.80	− 68.965	− 95.826
**s9**	− 2.407	− 61.924	− 89.332
**s10**	− 2.451	− 53.384	− 68.563
**s11**	− 2.613	− 65.022	− 97.947
**s12**	− 0.587	− 72.015	− 107.685
**s13**	− 3.313	− 74.499	− 93.797
**s14**	− 1.603	− 66.638	− 92.211
**s15**	− 2.159	− 57.488	− 88.326
**s16**	− 1.748	− 69.836	− 93.661
**GTP**	− 10.434	− 80.151	− 126.517

**Table 6:** Docking results of most active compounds **s3** and **s14** with GTP

Comp.	Docking score	Glide energy (kcal/mol)	Glide emodel	Interacting residues
**s3**	− 2.14	− 56.46	− 75.84	Thr35, Pro34, Asp33, Cys32, Glu31, Asp30, Phe28, Ser145, Ala146, Lys147, Leu120, Asp119, Lys117, Asn116, Asp57, Thr58, Ala59, Gly60, Ala18, Ser17, Lys16, Gly15, Gly13, Gly12
**s14**	− 1.603	− 66.638	− 92.211	Ala83, Asn85, Asn86, Lys117, Phe28, Val29, Asp30, Glu31, Cys32, Asp33, Pro34, Thr35, Asp57, Thr58, Ala59, Gly60, Gln61, Gly12, Gly13, Val14, Gly15, Lys16, Ser17, Ala18
**GTP**	− 10.434	− 80.151	− 126.517	Leu120, Asp119, Lys117, Asn116, Lys147, Ala146, Ser145, Phe28, Val29, Asp30, Glu31, Cys32, Asp33, Pro34, Thr35, Gly13, Gly15, Ser17, Ala18

### Anticancer screening results

Table 3 shows the comparison between HCT116 and RAW 264.7 of the IC_50_ values of the 1,3-diazine derivatives (**s1**–**s16**). 1,3-Diazine compounds showed good selectivity of compounds to the human colorectal cell line of carcinoma instead of the murine macrophages. The compounds of IC_50_ 1,3-diazine versus RAW 264.7 were all beyond the largest concentration tested. Among the compounds tested (on the RAW 264.7 line of murine macrophages), compounds **s11** and **s16** showed better potency against the cell line of murine macrophages. The control drug had an antiproliferative impact on both lines of the cell.

### Cell toxicity analysis

These were screened against ordinary human embryonic renal cell line (HEK-293) for the selectivity index calculation of the chosen compounds. Compounds have been dissolved into DMSO solution of 0.1%. The concentration of the compounds (2 µM, 4 µM, 6 µM, 8 µM and 10 µM) was diluted. The cells were incubated with these compounds for 24 h and at IC_50_ for growth inhibition of each researched compound, nearly 100% of HEK-293 cells were feasible. Results showed the important difference in viability after 24 h with (P < 0.01) between the treated test compound and the control cells (at zero concentration). The 50% of neurons were feasible at the chosen compounds lethal dose (LD_50_) of 8.55 to 8.18 µM. As we understand, the LD_50_ value greater than the IC_50_ will be the selectivity that meant that the compounds could have better safety for each of the six compounds as the IC_50_ is much smaller than the LD_50_ compounds. Each compound’s selectivity index suggested better safety for each compound (Table 7).

**Table 7:** Lethal dose (LD_50_) and selectivity index calculation of most active compounds

S. no.	Comp.	Lethal dose LD_50_	Cancer cell line HCT116 (IC_50_)	Selectivity index (LD_50_/IC_50_)
1.	**s3**	8.55	1.16	7.37
2.	**s9**	8.45	2.96	2.85
3.	**s13**	8.23	2.63	3.12
4.	**s14**	8.18	2.18	3.75
5.	**s15**	8.34	2.64	3.15
6.	**s16**	8.48	3.59	2.36

## Conclusion

Computational methods such as PharmMapper and molecular docking are cost-effective and time-saving instrument used respectively to determine target protein and generate docking data. GTPase HRas was discovered to be a target receptor among the top five scored protein to study the antiproliferative potential of more effective compounds **s3** and **s14**. In the GTPase HRas protein binding site, the further docking of 1,3-diazine compounds produced the docking poses of the most active compound and GTP used as positive control. In addition, compounds **s11** and **s16** showed stronger anti-cancer activity against the cell line of murine macrophages. The impact of most active compounds on the cell viability of non-cancerous HEK-293 cells has also been investigated in the current research. The findings showed a stronger selectivity index at the corresponding concentration of IC_50_ against the HEK 293 cell lines. Study proposed that after experimental assessment, the compound may be safer as an anticancer. 1,3-Diazine compounds (**s1**–**s16**) showed excellent selectivity of the compounds towards the cell line of human colorectal carcinoma instead of the murine macrophages. After further experimental validation, most active compounds may be safer to use. The research suggested that GTPase HRas protein with a stronger selectivity index could be the possible target protein of 1,3-diazine compounds.

## Additional files

**Additional file 1.** Web link for GTPase HRas protein.

**Additional file 2.** Docking results of the data set.

**Additional file 3.** Docking results, Pictorial presentation and Ligand interaction diagram of GTPas.

## Abbreviations

HCT116: human colorectal carcinoma116
RAW 264.7: murine macrophaga 264.7
PDB: Protein Data Bank
GTP\HR-protein: guanosine-5′-triphosphate/Harvey rat-protein
XP: extra precision
2D: 2 dimensional
3D: 3 dimensional
SRB: sulforhodamine B
MTT: 3-(4,5-dimethylthiazol-2-yl)-2,5-diphenyltetrazolium bromide
HEK-293: human embryonic kidney-293
SI: selectivity index
LD_50_: lethal dose_50_
5-Fu: 5-fluorouracil
RNA: ribonucleic acid
DNA: deoxyribonucleic acid
NMR: nuclear magnetic resonance
IR: infrared
MS: mass spectrum
CHN: carbon hydrogen nitrogen
Str: starching
