# Supplementary figures and images for: Computational approaches: discovery of GTPase HRas as prospective drug target for 1,3-diazine scaffolds
Source: BMC Chem. 2019 Jul 24;13(1):96. doi: 10.1186/s13065-019-0613-8 (PMC6659553; doi:10.1186/s13065-019-0613-8)

## Additional File 1

**Web link for GTPase HRas protein (PDB Id: 2CL7)**

<https://www.rcsb.org/>

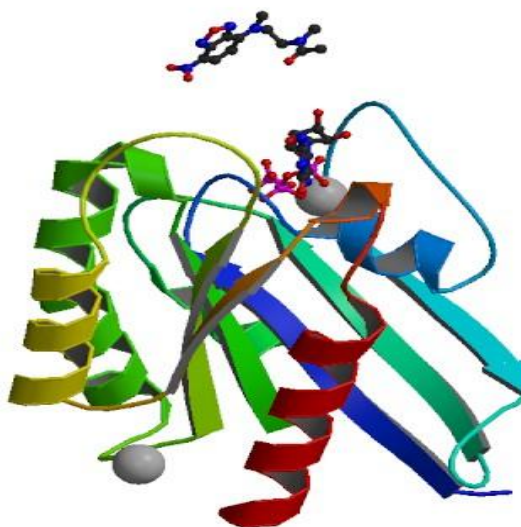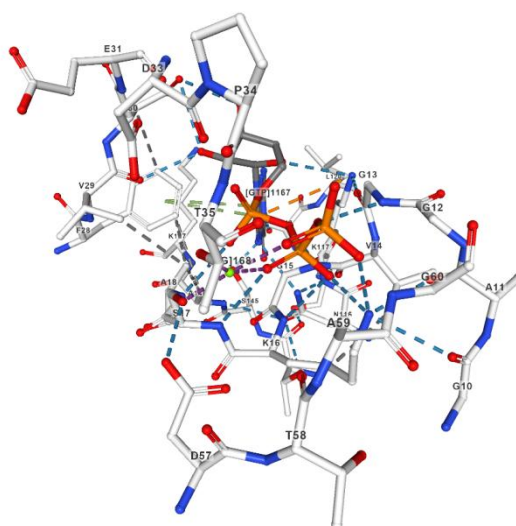

## Protein structure

Supplement: Supplementary file 1 — Additional file 1. Web link for GTPase HRas protein. [file 13065_2019_613_MOESM1_ESM.pdf]
