# Supplementary material for: Computational approaches: discovery of GTPase HRas as prospective drug target for 1,3-diazine scaffolds
Source: BMC Chem. 2019 Jul 24;13(1):96. doi: 10.1186/s13065-019-0613-8 (PMC6659553; doi:10.1186/s13065-019-0613-8)
Supplement: Supplementary file 2 — Additional file 2. Docking results of the data set. [file 13065_2019_613_MOESM2_ESM.pdf]

Additional File 2
DOCKING RESULTS OF THE DATA SET

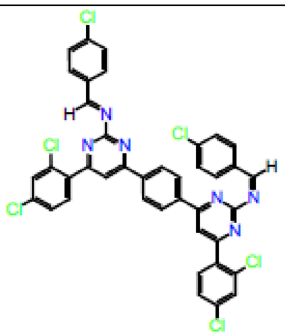<br>entry name: s1.1<br>docking score: -0.951<br>glide energy: -68.028<br>glide emodel: -93.889<br>Source File: glide dock_XP_1_pv.maegz	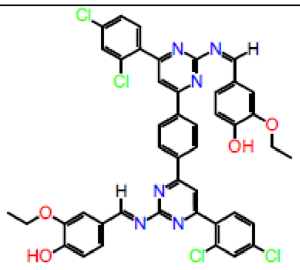<br>entry name: s2.1<br>docking score: -4.195<br>glide energy: -80.703<br>glide emodel: -107.394<br>Source File: glide dock_XP_1_pv.maegz	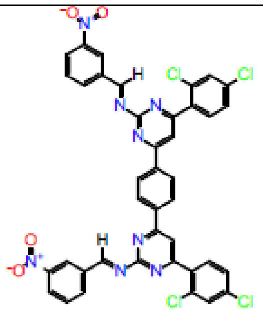<br>entry name: s3.1<br>docking score: -2.14<br>glide energy: -56.46<br>glide emodel: -75.84<br>Source File: glide dock_XP_1_pv.maegz
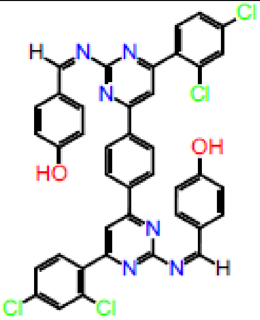<br>entry name: s4.1<br>docking score: -2.816<br>glide energy: -71.535<br>glide emodel: -99.49<br>Source File: glide dock_XP_1_pv.maegz	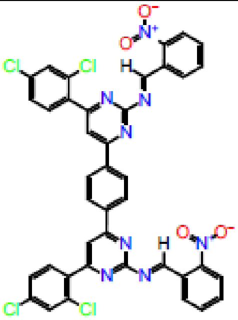<br>entry name: s5.1<br>docking score: -2.316<br>glide energy: -54.01<br>glide emodel: -72.719<br>Source File: glide dock_XP_1_pv.maegz	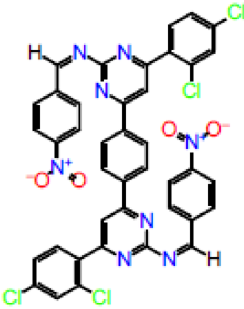<br>entry name: s6.1<br>docking score: -1.394<br>glide energy: -78.785<br>glide emodel: -112.194<br>Source File: glide dock_XP_1_pv.maegz
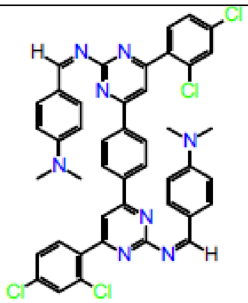<br>entry name: s7.1<br>docking score: -1.077<br>glide energy: -76.603<br>glide emodel: -109.074<br>Source File: glide dock_XP_1_pv.maegz	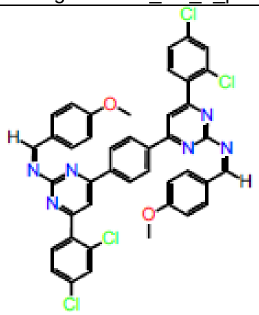<br>entry name: s8.1<br>docking score: -0.80<br>glide energy: -68.965<br>glide emodel: -95.826<br>Source File: glide dock_XP_1_pv.maegz	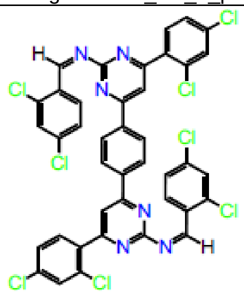<br>entry name: s9.1<br>docking score: -2.407<br>glide energy: -61.924<br>glide emodel: -89.332<br>Source File: glide dock_XP_1_pv.maegz
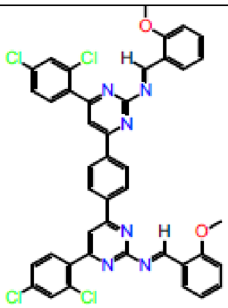<br>entry name: s10.1<br>docking score: -2.451<br>glide energy: -53.384<br>glide emodel: -68.563<br>Source File: glide dock_XP_1_pv.maegz	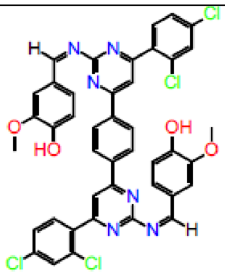<br>entry name: s11.1<br>docking score: -2.613<br>glide energy: -65.022<br>glide emodel: -97.947<br>Source File: glide dock_XP_1_pv.maegz	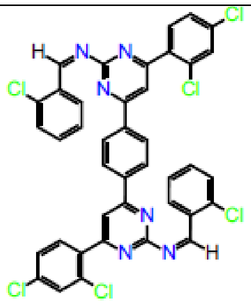<br>entry name: s12.1<br>docking score: -0.587<br>glide energy: -72.015<br>glide emodel: -107.685<br>Source File: glide dock_XP_1_pv.maegz

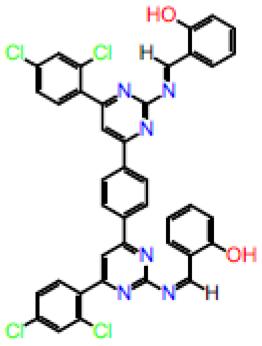

entry name: s13.1
docking score: -3.313
glide energy: -74.499
glide emodel: -93.797
Source File: glide dock_XP_1_pv.maegz

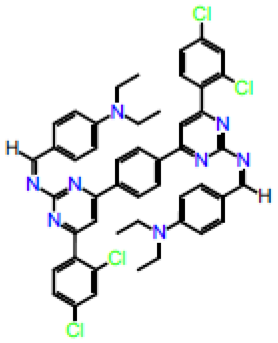

entry name: s14.1
docking score: -1.603
glide energy: -66.638
glide emodel: -92.211
Source File: glide dock_XP_1_pv.maegz

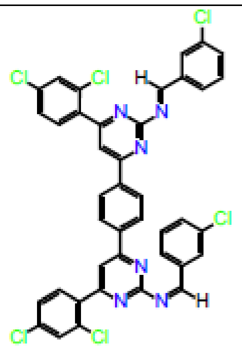

entry name: s15.1
docking score: -2.159
glide energy: -57.488
glide emodel: -88.326
Source File: glide dock_XP_1_pv.maegz

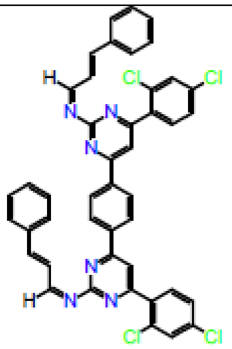

entry name: s16.1
docking score: -1.748
glide energy: -69.836
glide emodel: -93.661
Source File: glide dock_XP_1_pv.maegz
