# Supplementary material for: Computational approaches: discovery of GTPase HRas as prospective drug target for 1,3-diazine scaffolds
Source: BMC Chem. 2019 Jul 24;13(1):96. doi: 10.1186/s13065-019-0613-8 (PMC6659553; doi:10.1186/s13065-019-0613-8)
Supplement: Supplementary file 3 — Additional file 3. Docking results, Pictorial presentation and Ligand interaction diagram of GTPas. [file 13065_2019_613_MOESM3_ESM.pdf]

Additional File 3

Docking results, Pictorial presentation 3D and Ligand interaction diagram 2D of GTP

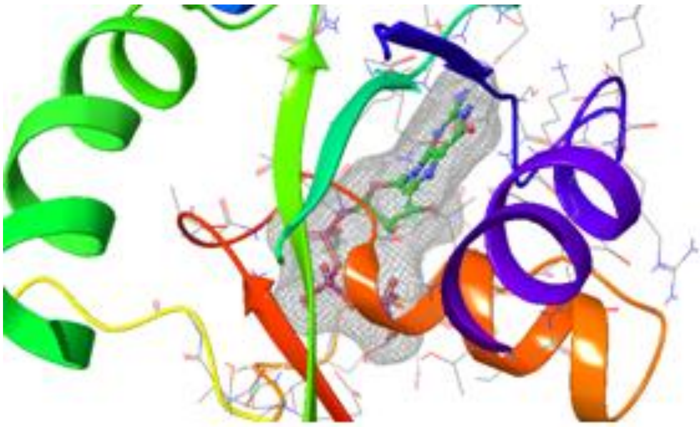

Pictorial presentation 3D

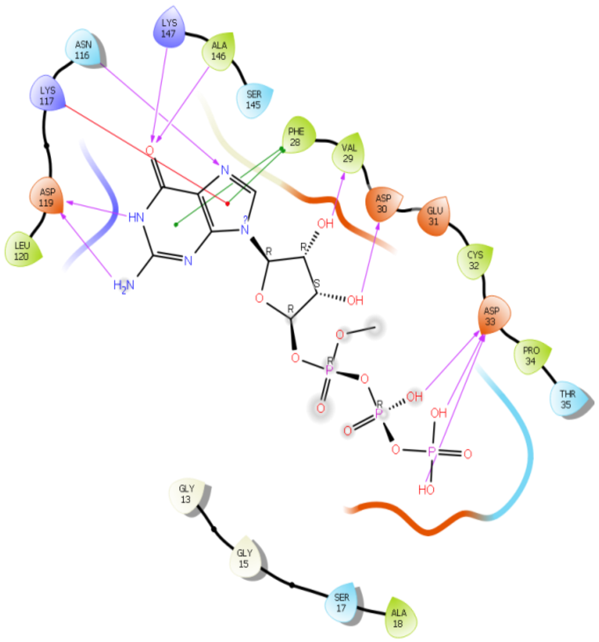

Ligand interaction diagram 2D

entry name: GTP
docking score: -10.434
glide energy: -80.151
glide emodel: -126.517
Source File: glide dock_XP_1_pv.maegz
